# Detection of zoonotic pathogens in invasive black rats (*Rattus rattus*) inside and outside a coastal protected area in southern Peru

**DOI:** 10.3389/fvets.2026.1750244

**Published:** 2026-05-29

**Authors:** Carlos Calvo-Mac, Wilmer Silva-Caso, Juana del Valle-Mendoza, Yordi Tarazona-Castro, Jessy Condori, Edith S. Malaga-Machaca, Maritza Calderón, Anne Martínez-Ventura, Pablo Tsukayama, Susana Cárdenas-Alayza

**Affiliations:** 1 Centro para la Sostenibilidad Ambiental, Universidad Peruana Cayetano Heredia (UPCH), Lima, Peru; 2 Laboratorio de Biomedicina, Centro de Investigación de la Facultad de Ciencias de la Salud, Universidad Peruana de Ciencias Aplicadas (UPC), Lima, Peru; 3 Laboratorio de Investigación en Enfermedades Infecciosas, Universidad Peruana Cayetano Heredia (UPCH), Lima, Peru; 4 Laboratorio de Genómica Microbiana, Facultad de Ciencias e Ingeniería, Universidad Peruana Cayetano Heredia (UPCH), Lima, Peru; 5 Departamento Académico de Ciencias Biológicas y Fisiológicas, Facultad de Ciencias e Ingeniería, Universidad Peruana Cayetano Heredia (UPCH), Lima, Peru

**Keywords:** apicomplexa, black rats, HPAI, leptospirosis, toxoplasmosis, zoonosis

## Abstract

**Introduction:**

This study investigates the occurrence of *Leptospira* spp., *Toxoplasma gondii*, and respiratory viruses in invasive black rats (*Rattus rattus*) from Punta San Juan (PSJ), a coastal protected area in southern Peru. Rodents can harbor zoonotic pathogens at the wildlife–human interface, posing ecological and public health risks.

**Methods:**

Fifty-three rats were trapped inside and outside PSJ in June 2025 from two contrasting zones: inside PSJ (*n* = 29), corresponding to the protected coastal habitat within the reserve, and outside PSJ (*n* = 24), including adjacent urban and human-impacted coastal areas. Serum, blood, and tissues were analyzed for *T. gondii* using Western blot and quantitative PCR. *Leptospira* spp. detection was performed by real-time PCR targeting the *lipL32* gene in blood samples. Respiratory viruses, including influenza A and B, were screened using the Illumina Respiratory Virus Panel through next-generation sequencing (NGS) technology in respiratory tract and lung swabs.

**Results:**

One adult female (1/49, 2.0%) captured inside PSJ was seropositive for *T. gondii*, but all PCR tests were negative in blood and tissues. *Leptospira* spp. DNA was detected in 18/53 rats (34.0%), with higher frequency outside the reserve (54.2%) than inside (17.2%) (*p* = 0.008). No amplifications were obtained for respiratory viruses.

**Discussion:**

The higher *Leptospira* frequency outside PSJ suggests that human-associated environments increase infection risk in the study area. In contrast, evidence for other zoonotic pathogens was limited, with only a single serological detection of *T. gondii* and no respiratory viruses identified. Although these findings are restricted to a local context, they highlight *Leptospira* spp. as the primary zoonotic pathogen detected and support the need for broader surveillance to better assess the epidemiological role of invasive rodents in coastal protected areas.

## 1 Introduction

Invasive rodents have had catastrophic effects on island and coastal ecosystems worldwide, particularly through their predation on seabirds and eggs, leading to population declines and local extinctions (1). Beyond their direct ecological impact, rodents also pose significant health risks by harboring zoonotic pathogens capable of infecting wildlife, domestic animals, and humans (2). Including species inhabiting coastal and marine ecosystems (3, 4).

Punta San Juan (PSJ), located in the Ica region of Peru, is part of the National Reserve System of Guano Islands, Islets, and Capes (Reserva Nacional Sistema de Islas, Islotes y Puntas Guaneras, RNIPG). This reserve system has hosted guano harvests which involve camps of over 100 workers that live for months at these sites to harvest or extract seabird guano that is then distributed as fertilizer. The protected area harbors remarkable biodiversity, including three residential marine mammal species: the South American sea lion (*Otaria byronia*), the South American fur seal (*Arctocephalus australis*), and the marine otter (*Lontra felina*). PSJ is also home to guano-producing seabirds the Guanay cormorant (*Phalacrocorax bougainvilllii*), the Peruvian booby (*Sula* var*iegata*) and the Peruvian pelican (*Pelecanus thagus*). It is also recognized as an important breeding colony for Humboldt penguins (*Spheniscus humboldti*), a globally threatened species (5). A perimeter 1.2 km long and 2.5 m high wall surrounding the reserve has been crucial in maintaining biodiversity by limiting human and domestic animal access. This physical barrier was built to protect the area from external pressures such as urban expansion from the nearby city (2 km) of San Juan de Marcona.

However, the presence of invasive rodents in Punta San Juan poses a serious threat to wildlife health, ecological balance, and public health. Black rats (*Rattus rattus*), which have colonized the area, negatively affect the ecosystem by preying on seabird eggs and chicks (6–8). *Rattus rattus* is a highly adaptable and opportunistic rodent species that thrives in coastal and insular environments, particularly in areas with seabird colonies and human influence. Its omnivorous diet, and close association with anthropogenic habitats facilitate frequent contact with wildlife, domestic animals, and contaminated environments, making it a relevant species for the circulation and maintenance of zoonotic pathogens at the terrestrial–marine interface (2, 9, 10).

*Toxoplasma gondii* is a protozoan parasite that causes toxoplasmosis in warm-blooded vertebrates and exhibits a complex life cycle involving both definitive and intermediate hosts (11). Felids are the definitive hosts, with domestic cats (*Felis catus*) being the primary host species responsible for the global spread of the parasite (12, 13). Wildlife may become infected through ingestion of infected prey, or by contact with contaminated water, soil, or fecal matter from infected felids (14, 15). In intermediate hosts such as rodents, *T. gondii* can establish in multiple tissues, particularly in the brain and heart, forming cysts that can persist throughout the host’s lifetime (16). In wildlife populations, toxoplasmosis can cause sublethal effects such as reproductive disorders (17) or contribute to population weakening, increasing vulnerability to stochastic environmental events (18). Aquatic animals are particularly susceptible to *T. gondii* exposure, as toxoplasmosis is a waterborne disease and oocysts can be transported through freshwater and marine runoff (11, 19).

Similarly, the genus *Leptospira*, is a globally distributed spiroqueta that can cause leptospirosis in humans and a wide range of animal species (20). These bacteria infect mammals, birds, and reptiles, with both domestic and wild rodents, carnivores, and ruminants serving as important reservoirs (21). Pathogenic *Leptospira* are shed in the urine of infected hosts, contaminating soil and water, and are efficiently transmitted in aquatic or humid environments (22). Exposure to *Leptospira* has been reported in numerous wildlife species, particularly in aquatic animals, as leptospirosis is primarily a waterborne disease (23, 24). The risk of transmission is especially high in areas with poor sanitation, agricultural activity, and urban development, where contact between humans, domestic animals, and wildlife is more frequent (25).

The outbreak of highly pathogenic avian influenza (HPAI) A(H5N1) in November 2022 in Peru, which affected both marine and terrestrial species, including seabirds, marine mammals, and poultry nationwide, resulted in significant mortality among sea lions and seabirds (26). Punta San Juan and its surrounding areas were not spared from this epizootic event, as both avian and marine mammal populations were affected (27–29). Experimental and field evidence further indicates that wild and synanthropic rodents, such as *R. rattus* and *R. norvegicus*, can harbor and replicate avian-origin H5N1 viruses in respiratory tissues, suggesting that they may act as replication-competent hosts and contribute to viral maintenance in human-dominated environments (30–34). The proximity of Punta San Juan to urban areas, where domestic animals such as cats, dogs, and livestock are present, represents a significant risk factor for the introduction and transmission of these and other zoonotic pathogens.

Although rodents are widely recognized as hosts of zoonotic pathogens, information from coastal protected areas in southern Peru is virtually absent. In particular, no studies have evaluated pathogen occurrence in *R. rattus* populations inhabiting the interface between a marine protected area and nearby urban environments. Therefore, the present study aims to evaluate the occurrence and detection frequency of selected zoonotic pathogens, including respiratory virus such as influenza A, *T. gondii*, and *Leptospira* spp. in black rats (*Rattus rattus*) captured across two contrasting areas: within Punta San Juan (protected area) and in adjacent urban and coastal zones.

## 2 Materials and methods

### 2.1 Study area and design

The study was conducted in and around Punta San Juan (PSJ), located in the Ica region of southern Peru (15°22′S, 75°11′W). A cross-sectional comparative study design was adopted to evaluate *Rattus rattus* populations across two zones with contrasting ecological and anthropogenic characteristics: (i) inside Punta San Juan (PSJ): comprises the coastal habitat within the perimeter wall of the reserve; (ii) outside Punta San Juan (urban and coastal zone): This area includes sites located more than 0.5 km beyond the perimeter wall, encompassing zones of intense human activity, such as tourist beaches, a marble quarry, and informal waste disposal sites. It also includes the city of San Juan de Marcona, characterized by elevated levels of anthropogenic pressure and frequent interactions among humans, domestic animals (dogs, cats, poultry, and livestock), and wildlife.

### 2.2 Ethical statement

This study was conducted in accordance with relevant national legislation and institutional guidelines for the use of animals in research. Fieldwork and sample collection were authorized under research permits RJ N° 000008-2024-SERNANP/RNIPG-SGD and RJ N° 000008-2025-SERNANP/RNIPG-SGD issued by the National Service of Natural Protected Areas (SERNANP), and RD N° D00033-2025-MIDAGRI-SERFOR-DGGSPFFS-DGSPFS issued by the National Forest and Wildlife Service (SERFOR), Peru. All animal handling and sampling procedures were approved by the Institutional Animal Care and Use Committee of Universidad Peruana Cayetano Heredia (CIEA-UPCH: CONSTANCIA-CIEA-038-08-24; CONSTANCIA-CIEA-E-035-12-24; CONSTANCIA-CIEA-R-013-02-25).

### 2.3 Sample collection and processing

Black rats were captured inside and outside Punta San Juan during June 2025 using Tomahawk live traps baited with oatmeal, peanut butter and vanilla extract. Within the reserve, four Sherman traps were also used to increase capture efficiency. Trapping effort totaled 234 trap-nights, with 67 active traps inside the reserve (117 trap-nights) and 78 outside (117 trap-nights). Capture success was 23.9% (28/117) inside and 20.5% (24/117) outside the reserve, yielding 53 *Rattus rattus* in total, including two individuals captured in a single Tomahawk trap. Traps were checked early each morning, and captured animals were transported to a field laboratory for examination.

Animals were euthanized using a CO₂ chamber, in accordance with American Veterinary Medical Association (35) guidelines. Each individual was then sexed, aged (juvenile/adult), and measured for body weight and morphometric variables (total length, body, tail, hind foot, and ear). All procedures were carried out in accordance with biosafety and field handling recommendations outlined by the PREDICT Consortium (36).

Blood was collected by cardiac puncture using sterile syringes and divided into serum, whole blood with EDTA, and coagulated fractions. Blood samples were centrifuged at 3,500 × g for 10 min to separate serum for serological testing. Heart and brain tissues were collected for molecular analysis and preserved in 70% ethanol. Respiratory tract and lung swabs were obtained by making a small incision in the trachea and placed in Universal Transport Medium (UTM).

All samples requiring freezing, including serum, whole blood with EDTA, coagulated fractions and UTM swabs, were stored at −20 °C in the field between 4 to 16 days, transported on dry ice to Lima, and subsequently kept at −80 °C until processing, a maximum of 4 months after. Heart and brain tissues preserved in ethanol were maintained at room temperature during both storage and transport to the laboratory.

Different biological samples were collected to target each investigated pathogen based on their known tissue tropism and recommended diagnostic approaches. Serum samples were used for serological detection of *T. gondii* exposure (37, 38). Whole blood (EDTA) was collected for molecular detection of *Leptospira* spp., as leptospires circulate in blood during infection (39, 40). Kidney tissue and urine samples, which are required to assess renal colonization and shedding of *Leptospira* spp., were not analyzed in this study. Therefore, molecular detection was limited to blood samples and aimed at identifying active or recent infection rather than chronic carriage or shedding status. Blood clots, brain, and heart tissues were collected for molecular confirmation of *T. gondii*, given the parasite’s affinity for neural and muscular tissues (41). Respiratory tract and lung swabs were obtained for molecular screening of influenza A and other respiratory viruses, as these pathogens primarily replicate in the respiratory epithelium (30, 33).

### 2.4 Pathogen detection

For molecular detection of influenza A and other respiratory viruses, RNA was extracted from respiratory tract and lung swabs using the QIAamp Viral RNA Mini Kit (Qiagen, Germany) following the manufacturer’s instructions. Extracted RNA samples were analyzed using the Illumina Respiratory Virus Oligo Panel (Illumina, San Diego, CA, United States), which allows targeted enrichment and detection of more than 40 respiratory viruses, including influenza A and B, coronaviruses, adenoviruses, parainfluenza viruses, and metapneumovirus (42). This approach allowed broad, unbiased screening of respiratory viruses using next-generation sequencing (NGS).

Serological detection of *T. gondii* was carried out using Western blot analysis on serum samples, following the procedures described by Saavedra and Ortega (38) and Flores et al. (37). For molecular confirmation, DNA was extracted from blood clot, brain, and heart tissues using a High Pure PCR Template Preparation Kit (Roche Diagnostics Corp., Indianapolis, IN) according to the manufacturer’s instructions. Quantitative PCR (qPCR) targeting the REP529 repetitive element of *T. gondii* was subsequently performed on these samples, following the protocol of Gutiérrez-Loli et al. (41).

For detection of *Leptospira* spp., DNA was extracted from whole blood samples using the High Pure PCR Template Preparation Kit (Roche Applied Science, Mannheim, Germany) according to the manufacturer’s instructions. Pathogenic *Leptospira* spp. were detected by real-time PCR targeting the lipL32 gene, following the protocols described by Stoddard et al. (43) and Silva-Caso et al. (40).

### 2.5 Statistical analyses

Detection frequency for each pathogen was calculated as the proportion of positive individuals among the sampled rats, with 95% confidence intervals (CI). A principal component analysis (PCA) was first performed on standardized morphometric variables (body weight, total length, tail length, ear, and hind foot) to generate a body size index (PC1) representing overall body size. Body size was included as a proxy for age and cumulative exposure, as larger and older rodents are more likely to have experienced repeated contact with contaminated environments and pathogens, potentially increasing their probability of infection (44, 45). Associations between infection status and categorical variables (sex, age, and sampling zone) were evaluated using Fisher’s exact tests, while comparisons involving the body size index (PC1) were assessed using the Mann–Whitney U test. A multivariable logistic regression model was then fitted to identify predictors of infection, including sampling zone (inside vs. outside the reserve), sex, age, and body size index. Odds ratios (OR) and 95% confidence intervals were estimated, and statistical significance was set at *p* < 0.05. All statistical analyses were conducted using R software (version 4.1.1 (46);) with the packages epiR (47), FactoMineR (48), and stats (46).

## 3 Results

A total of 53 *Rattus rattus* individuals were analyzed, with different biological samples tested according to the diagnostic target of each pathogen (serum for *T. gondii* serology; blood for molecular detection of *Leptospira* spp.; respiratory tract and lung swabs for respiratory viruses).

No amplifications were obtained for respiratory viruses in any respiratory tract or lung swab. Serological testing for *T. gondii* was performed on 49 serum samples, as insufficient blood was obtained from four individuals. One adult female (1/49, 2.0, 95% CI, 0.1–10.7%), captured inside the reserve, tested positive for *T. gondii* IgG antibodies. Molecular analysis of this individual’s blood clot, heart, and brain tissues yielded negative results by qPCR. Subsequently, brain samples from an additional 24 individuals were tested by qPCR targeting the REP529 gene, and all were negative for *T. gondii* DNA.

**Figure 1:**
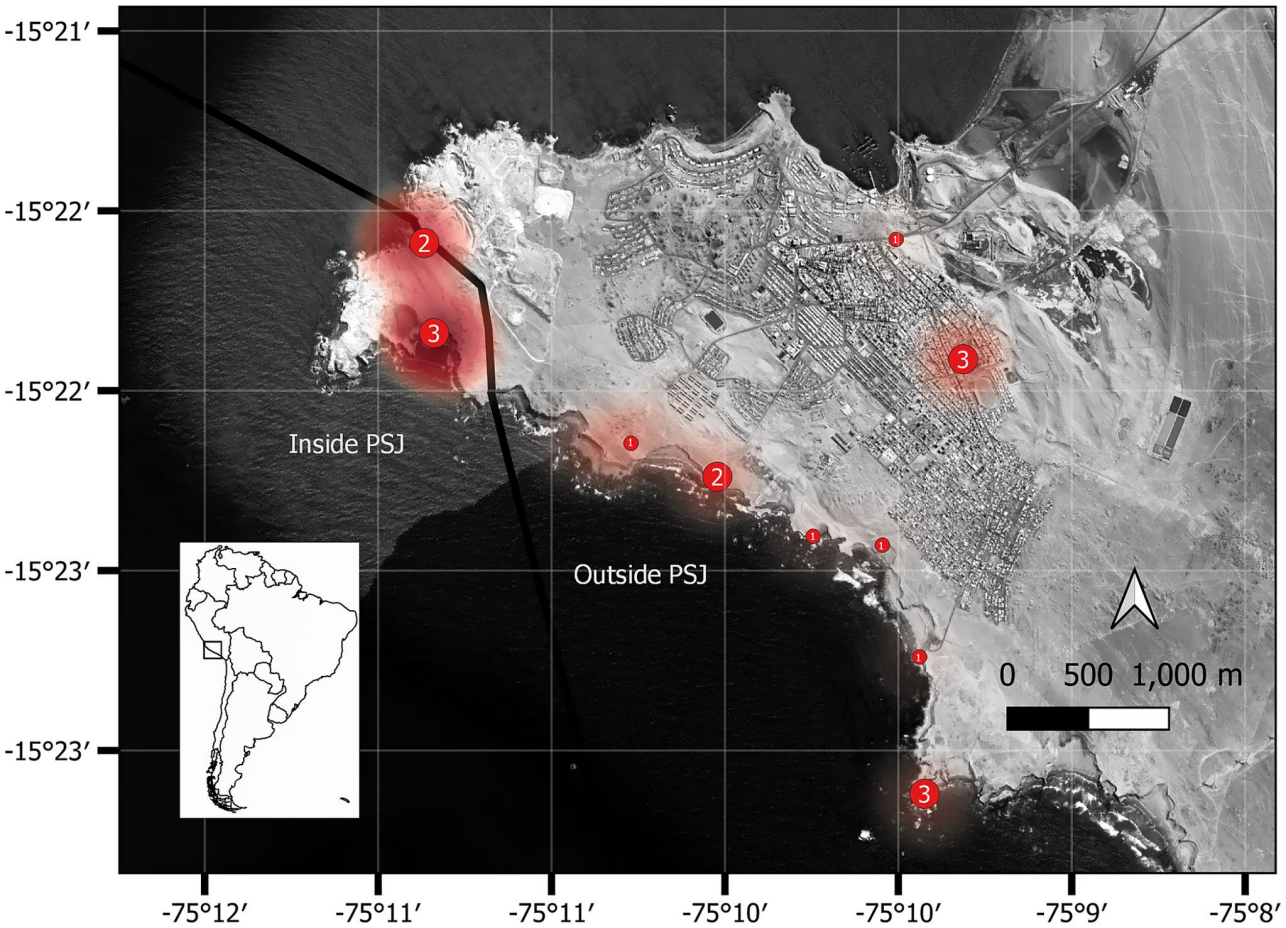
Study area of Punta San Juan (PSJ) and surrounding coastal zones in southern Peru. Red heat maps show the sampling effort in the entire study area. Circles with numbers show the PCR-positive detections for *Leptospira* spp. in black rats (*Rattus rattus*).

*Leptospira* spp. DNA was detected in 18 of 53 rats’ blood samples (34.0%; 95% CI: 21.2–48.8%) (Table 1; Figure 1). The proportion of positives was significantly higher outside the reserve (13/24; 54.2, 95% CI: 33.2–73.7%) than inside (5/29, 17.2, 95% CI: 7.6–34.5%), according to Fisher’s exact test (*p* = 0.008). No significant differences in infection status of *Leptospira* spp. were observed between sexes, age classes, or body size index (PC1) values. In the multivariable logistic regression model, sampling zone (inside vs. outside the reserve) was the only significant predictor of infection by *Leptospira* spp. (OR = 7.3, 95% CI: 1.9–28.1, *p* = 0.004).

**Table 1:** Molecular detection of *Leptospira* spp. by qPCR in invasive black rats (*Rattus rattus*) from Punta San Juan (PSJ) and surrounding coastal areas, southern Peru.

Variable	Positive/total (%)	95% CI or mean ± SD^a^	*p*-value
Sex			0.565 ^b^
Male	6/21 (28.6)	11.3–52.2	
Female	12/32 (37.5)	21.1–56.3	
Age class			0.83 ^b^
Adult	11/31 (35.5)	19.2–54.6	
Juvenile	7/22 (31.8)	14.7–54.9	
Zone			0.008 ^b^
Inside PSJ	5/29 (17.2)	7.6–34.5	
Outside PSJ	13/24 (54.2)	33.2–73.7	
Body size index (PCA1)			0.324 ^c^
Positive		0.290 ± 1.748	
Negative		−0.149 ± 1.989	
Overall	18/53 (34.0)	21.2–48.8	

^a^CI, Confidence interval; SD, Standard deviation; ^b^Fisher’s exact tests; ^c^Mann–Whitney U test.

## 4 Discussion

This study provides new insights into the occurrence of *Leptospira* spp. and *T. gondii* in invasive *Rattus rattus* populations from Punta San Juan (PSJ) and the surrounding coastal areas in southern Peru. Previous health assessments of marine fauna in PSJ reported no serological evidence of exposure to *T. gondii* or *Leptospira* spp. in South American fur seals (*Arctocephalus australis*) (49). However, these assessments were conducted several years ago, and the epidemiological context may have changed substantially. Recent studies in Chile have reported seropositivity to both pathogens in pinnipeds, particularly in individuals that inhabit areas with greater human activity or domestic animal presence (50, 51). Both *Leptospira* and *T. gondii* can cause morbidity and mortality in marine mammals, including pinnipeds (52, 53), and infected rodents near reproductive colonies of fur seals and/or sea lions could represent a potential source of pathogen spillover (54, 55). Moreover, pinnipeds themselves may act as carriers and play an important role in the maintenance and transmission of *Leptospira* spp. within coastal ecosystems (56). Chronic shedding of *Leptospira interrogans* in California sea lions (*Zalophus californianus*) has been shown to sustain bacterial persistence between outbreaks, enabling long-term maintenance of infection within populations despite seasonal variation in transmission (57).

Surveys of *T. gondii* and *Leptospira* spp. exposure have not yet been conducted in seabird populations of PSJ, including the Humboldt penguin (*Spheniscus humboldti*), Guanay cormorant (*Leucocarbo bougainvillii*), and Peruvian pelican (*Pelecanus thagus*) (58, 59). Further investigation into these species is warranted given their ecological overlap with terrestrial mammals, the presence of invasive rodents, and their potential exposure to contaminated environments. In other coastal regions, serological evidence of *T. gondii* infection has been reported in seabirds (60–62), and fatal toxoplasmosis has been documented in penguins (63, 64). In contrast, information on *Leptospira* infection in birds remains scarce. Although exposure to *Leptospira* spp. has been reported in two Magellanic penguins (*Spheniscus magellanicus*) and other wild birds in Chile (50), other studies from Chile and Brazil have failed to detect exposure in other seabird species (60, 61). Thus, the epidemiological role of Peruvian seabirds in *Leptospira* transmission, their potential contribution to pathogen circulation, and the clinical significance of infection remain unknown.

The detection of *T. gondii* in a single rodent inside the reserve and the higher molecular detection frequency of *Leptospira* spp. infection in black rats captured outside PSJ suggests distinct ecological and epidemiological dynamics of pathogens associated with habitat type and human influence. These findings highlight the potential involvement of *Rattus rattus* in pathogen circulation at the interface between wildlife, domestic animals, and humans in coastal ecosystems (20). Because *Leptospira* detection was based on PCR amplification of bacterial DNA in blood, these results reflect active or recent infection (39). Therefore, the present findings demonstrate ongoing circulation of pathogenic *Leptospira* among black rats in the study area, but do not allow inference on chronic carriage, shedding dynamics, or long-term reservoir competence.

Leptospira detection was reported at the genus level, as the real-time PCR protocol used targets the lipL32 gene, which is conserved among pathogenic *Leptospira* species (40, 43). While molecular typing or serovar identification would provide higher epidemiological resolution, such analyses were beyond the scope of this study and require additional molecular markers, isolation, or sequencing approaches. Future studies incorporating genotyping or multilocus sequence typing would substantially enhance understanding of transmission pathways and public health relevance.

The presence of *Leptospira*-positive black rats in areas surrounding the reserve indicates the possible introduction of pathogenic strains from nearby urban zones, where synanthropic rats thrive and may facilitate pathogen spillover into protected habitats (65). *Leptospira* can persist for extended periods in humid environments and be transmitted through urine-contaminated soil or water, posing infection risks for both wildlife and humans (22). Consequently, its detection on beaches adjacent to the reserve represents a direct occupational hazard for local fishers and kelp collectors, who often work barefoot or in close contact with seawater, kelp, caves, sand and gravel on the beach. Moreover, as marine mammals and seabirds frequently congregate in these areas, cross-species transmission events cannot be ruled out, underscoring the need for coordinated pathogen monitoring under a One Health framework.

The absence of viral RNA in the respiratory samples analyzed does not necessarily exclude rodent participation in viral maintenance or transmission but rather provides an essential baseline for future surveillance. Considering the increasing diversity of respiratory and zoonotic viruses recently identified in wild rodent populations worldwide (66, 67), continued metagenomic screening in coastal ecosystems of South America is warranted to better elucidate the viral communities circulating in invasive species and their potential for interspecies transmission to inform and promote eradication or removal of these reservoirs from sensitive wild areas.

This study has several limitations that should be considered when interpreting the results. The sample size was relatively small, which may have limited the ability to detect statistical associations. An additional limitation of this study is the absence of serological testing for *Leptospira* spp., which precludes assessment of past exposure and population-level prevalence. Furthermore, leptospiral persistence and shedding are primarily associated with renal colonization; therefore, future studies should include molecular and histopathological analyses of kidney tissue, and ideally urine samples, to confirm renal carriage and assess chronic infection status and reservoir potential in invasive rodent populations (39, 68, 69). In the urban area outside the reserve, trapping success was constrained by low public acceptance of rodent capture and the abundance of alternative food sources reduced bait efficiency. The presence of free-roaming domestic cats in the city may also have influenced rodent activity and spatial distribution (70).

Beyond the influence of urbanization, environmental conditions likely play a critical role in *Leptospira* persistence and transmission. Accumulation of garbage, organic waste, and informal dumpsites in the desert and on polluted beaches around PSJ creates microhabitats that favor rodent proliferation and bacterial survival in moist substrates (70). Seasonal and longitudinal sampling would also help reveal temporal fluctuations in *Leptospira* circulation and identify environmental drivers of infection. Expanding surveillance to include domestic animals, livestock, and local human populations would further clarify the ecological dynamics of leptospirosis in the area (25). Integrating these approaches under a One Health perspective will be essential for identifying transmission pathways and developing effective mitigation strategies.

Overall, these findings emphasize the importance of sustained surveillance and rodent management at the human–wildlife interface in coastal protected areas. Continuous monitoring of invasive species, combined with wildlife and environmental pathogen surveillance, will support early detection of emerging diseases and mitigate health risks to both biodiversity and local communities.

## Data Availability

The data presented in this study are included in the article and its supplementary material. Further inquiries can be directed to the corresponding author.

## References

[1] Towns DR Byrd GV Jones HP Rauzon MJ Russell JC Wilcox C. "Impacts of introduced predators on seabirds". In: Mulder CPH Anderson WB Towns DR Bellingham PJ, editors. Seabird Islands: Ecology, Invasion, and Restoration. New York: Oxford University Press (2011). p. 56–90.

[2] Dahmana H Granjon L Diagne C Davoust B Fenollar F Mediannikov O. Rodents as hosts of pathogens and related zoonotic disease risk. Pathogens. (2020) 9:202. doi: 10.3390/pathogens9030202, 32164206 PMC7157691

[3] Gulland FM Hall AJ. Is marine mammal health deteriorating? Trends in the global reporting of marine mammal disease. EcoHealth. (2007) 4:135–50. doi: 10.1007/s10393-007-0097-1

[4] Vanstreels RET Uhart MM Work TM. "Health and Diseases". In: González-Solís JA Oro D, editors. Conservation of Marine Birds. San Diego, CA: Academic Press (2023). p. 131–76.

[5] Goya Sueyoshi E Bachmann Caller VM Llapapasca Lloclla M de Márquez Manrique Lara JC Meza Torres MA Quiñones Dávila J . Depredadores superiores en Punta San Juan, Perú, GEF UNDP. 2014. Inf Inst Mar Perú. (2020) 47:96–121.

[6] Ampuero-Merino L Colchao P Nishio F Cárdenas-Alayza S. *Monitoreo de la Comunidad de Roedores (Rattus rattus) en la Reserva Punta San Juan, Parte de la Reserva Nacional Sistema de Islas, Islotes y Puntas Guaneras (RNSIIPG)*. Programa Punta San Juan, Centro para la Sostenibilidad Ambiental, Universidad Peruana Cayetano Heredia, p. 35. (2015).

[7] Ampuero-Merino L Cárdenas-Alayza S. *Impacto de Depredadores Introducidos Sobre la Población Reproductiva de Zarcillos (Larosterna inca) en Punta San Juan, Ica, Perú*. Abstract retrieved from V Congreso de Ciencias del Mar del Perú. Lambayeque, Perú (2016).

[8] Ampuero-Merino L Alfaro-Córdova E Alfaro-Shigueto J. *Primer Reporte de Depredación de Roedores en Piqueros y las Implicancias Para la Conservación de Aves Marinas en las Islas Lobos de Afuera*. Abstract retrieved from IV Congreso de Ciencias del Mar del Perú. Huacho, Perú. (2018).

[9] Boey K Shiokawa K Rajeev S. *Leptospira* infection in rats: a literature review of global prevalence and distribution. PLoS Negl Trop Dis. (2019) 13:e0007499. doi: 10.1371/journal.pntd.0007499, 31398190 PMC6688788

[10] Shiels AB Pitt WC Sugihara RT Witmer GW. Biology and impacts of Pacific Island invasive species 11. The black rat, *Rattus rattus* (Rodentia: Muridae). Pac Sci. (2014) 68:11.

[11] Jones JL Dubey JP. Waterborne toxoplasmosis–recent developments. Exp Parasitol. (2010) 124:10–25. doi: 10.1016/j.exppara.2009.03.013, 19324041

[12] Innes EA. A brief history and overview of *Toxoplasma gondii*. Zoonoses Public Health. (2010) 57:1–7. doi: 10.1111/j.1863-2378.2009.01276.x, 19744303

[13] Lehmann T Marcet PL Graham DH Dahl ER Dubey JP. Globalization and the population structure of *Toxoplasma gondii*. Proc Natl Acad Sci U S A. (2006) 103:11423–8. doi: 10.1073/pnas.0601438103, 16849431 PMC1544101

[14] Aguirre AA Longcore T Barbieri M Dabritz H Hill D Klein PN . The one health approach to toxoplasmosis: epidemiology, control, and prevention strategies. EcoHealth. (2019) 16:378–90. doi: 10.1007/s10393-019-01405-7 30945159 PMC6682582

[15] Shapiro K Bahia-Oliveira L Dixon B Dumètre A de Wit LA VanWormer E . Environmental transmission of *Toxoplasma gondii*: oocysts in water, soil and food. Food Waterborne Parasitol. (2019) 15:e00049. doi: 10.1016/j.fawpar.2019.e00049, 32095620 PMC7033973

[16] Galeh TM Sarvi S Montazeri M Moosazadeh M Nakhaei M Shariatzadeh SA . Global status of *Toxoplasma gondii* seroprevalence in rodents: a systematic review and meta-analysis. Front Vet Sci. (2020) 7:461. doi: 10.3389/fvets.2020.00461, 32851037 PMC7411222

[17] Formenti N Trogu T Pedrotti L Gaffuri A Lanfranchi P Ferrari N. *Toxoplasma gondii* infection in alpine red deer (*Cervus elaphus*): its spread and effects on fertility. PLoS One. (2015) 10:e0138472. doi: 10.1371/journal.pone.0138472, 26405785 PMC4583299

[18] Lafferty KD Gerber LR. Good medicine for conservation biology: the intersection of epidemiology and conservation theory. Conserv Biol. (2002) 16:593–604. doi: 10.1046/j.1523-1739.2002.00446.x

[19] Shapiro K Miller M Mazet J. Temporal association between land-based runoff events and California Sea otter (*Enhydra lutris nereis*) protozoal mortalities. J Wildl Dis. (2012) 48:394–404. doi: 10.7589/0090-3558-48.2.394, 22493114

[20] Antoniolli A Guis H Picardeau M Goarant C Flamand C. One health field approach applied to leptospirosis: a systematic review and meta-analysis across humans, animals and the environment. Open Forum Infect Dis. (2025) 12:ofae757. doi: 10.1093/ofid/ofae757, 39845019 PMC11752865

[21] Andersen-Ranberg EU Pipper C Jensen PM. Global patterns of *Leptospira* prevalence in vertebrate reservoir hosts. J Wildl Dis. (2016) 52:468–77. doi: 10.7589/2014-10-245, 27187029

[22] Bradley EA Lockaby G. Leptospirosis and the environment: a review and future directions. Pathogens. (2023) 12:1167. doi: 10.3390/pathogens12091167, 37764975 PMC10538202

[23] Bierque E Thibeaux R Girault D Soupé-Gilbert ME Goarant C. A systematic review of *Leptospira* in water and soil environments. PLoS One. (2020) 15:e0227055. doi: 10.1371/journal.pone.0227055, 31986154 PMC6984726

[24] Mapham PH Vorster JH. Leptospirosis in wildlife. Vetdiagnostix Vet Pathol. (2016) 20:12–6.

[25] Ullmann LS Langoni H. Interactions between environment, wild animals and human leptospirosis. J Venom Anim Toxins Incl Trop Dis. (2011) 17:119–29. doi: 10.1590/S1678-91992011000200002

[26] Leguia M Garcia-Glaessner A Muñoz-Saavedra B Juarez D Barrera P Calvo-Mac C . Highly pathogenic avian influenza a (H5N1) in marine mammals and seabirds in Peru. Nat Commun. (2023) 14:5489. doi: 10.1038/s41467-023-41182-0, 37679333 PMC10484921

[27] Allender MC Adkesson MJ Wang L Sheldon J Hemby C Ampuero-Merino LP . *Epidemiology of Highly Pathogenic Avian Influenza and Coronavirus in a Well-Monitored Marine Reserve in Peru*. Abstract retrieved from Abstracts in 2024 Annual Meeting of the American Association of Zoo Veterinarians, Toronto, Ontario, Canada (2024).

[28] Cárdenas-Alayza S Adkesson MJ Sheldon J Hemby C Ampuero-Merino LP Torres DA . *Multispecies Outbreak of Highly Pathogenic Avian Influenza in a Well-Monitored Marine Reserve in Peru*. Abstract retrieved from the 71st Annual International Conference of the Wildlife Disease Association, Athens, Georgia, USA (2023).

[29] Hemby CM Allender MC Cárdenas-Alayza S Kayastha S Wang L Sheldon JD. Comparison of rapid antigen test and quantitative PCR for detection of highly pathogenic avian influenza virus in free-ranging Peruvian seabirds. J Wildlife Dis. (2025) 61:756–9. doi: 10.7589/JWD-D-24-00188, 40405422

[30] Hatta M Hatta Y Kim JH Watanabe S Shinya K Nguyen T . Growth of H5N1 influenza a viruses in the upper respiratory tracts of mice. PLoS Pathog. (2007) 3:e133. doi: 10.1371/journal.ppat.0030133, 17922570 PMC2000968

[31] Kutkat O Gomaa M Moatasim Y El Taweel A Kamel MN El Sayes M . Highly pathogenic avian influenza virus H5N1 clade 2.3. 4.4 b in wild rats in Egypt during 2023. Emerg Microbes Infect. (2024) 13:2396874. doi: 10.1080/22221751.2024.2396874, 39193629 PMC11382695

[32] Li L Chen R Yan Z Cai Q Guan Y Zhu H. Experimental infection of rats with influenza a viruses: implications for murine rodents in influenza a virus ecology. Viruses. (2025) 17:495. doi: 10.3390/v17040495, 40284938 PMC12030792

[33] Usui T Uno Y Tanaka K Tanikawa T Yamaguchi T. Susceptibility of synanthropic rodents (*Mus musculus*, *Rattus norvegicus* and *Rattus rattus*) to H5N1 subtype high pathogenicity avian influenza viruses. Pathogens. (2024) 13:764. doi: 10.3390/pathogens13090764, 39338955 PMC11434905

[34] Velkers FC Blokhuis SJ Veldhuis Kroeze EJ Burt SA. The role of rodents in avian influenza outbreaks in poultry farms: a review. Vet Q. (2017) 37:182–94. doi: 10.1080/01652176.2017.1325537, 28460593

[35] American Veterinary Medical Association. AVMA Guidelines for the Euthanasia of Animals: 2020 Edition. Schaumburg, IL: American Veterinary Medical Association (2020).

[36] PREDICT Consortium. PREDICT Standard Operating Procedures for One Health Surveillance. Davis: One Health Institute, University of California (2020).

[37] Flores CA Jimenez J Gomez-Puerta LA Palacios C O'Neal SE Muro C . Seroprevalence of *Toxoplasma gondii* in free-range pigs in northern Peru. Vet Parasitol Reg Stud Rep. (2021) 23:100533. doi: 10.1016/j.vprsr.2021.100533, 33678386 PMC9125792

[38] Saavedra GM Ortega YR. Seroprevalence of *Toxoplasma gondii* in swine from slaughterhouses in Lima, Peru, and Georgia, USA. J Parasitol. (2004) 90:902–4. doi: 10.1645/GE-258R 15357100

[39] Adler B de la Peña Moctezuma A. *Leptospira* and leptospirosis. Vet Microbiol. (2010) 140:287–96. doi: 10.1016/j.vetmic.2009.03.012, 19345023

[40] Silva-Caso W Aguilar-Luis MA Espinoza-Espíritu W Vilcapoma-Balbin M Del Valle LJ Misaico-Revate E . *Leptospira* spp. and Rickettsia spp. as pathogens with zoonotic potential causing acute undifferentiated febrile illness in a central-eastern region of Peru. BMC Res Notes. (2024) 17:171. doi: 10.1186/s13104-024-06837-1, 38902784 PMC11188165

[41] Gutierrez-Loli R Ferradas C Diestra A Traianou A Bowman N Bok J . Development of a novel protocol based on blood clot to improve the sensitivity of qPCR detection of *Toxoplasma gondii* in peripheral blood specimens. Am J Trop Med Hyg. (2019) 100:83–9. doi: 10.4269/ajtmh.17-0920, 30457102 PMC6335924

[42] Sahajpal NS Mondal AK Njau A Petty Z Chen J Ananth S . High-throughput next-generation sequencing respiratory viral panel: a diagnostic and epidemiologic tool for SARS-CoV-2 and other viruses. Viruses. (2021) 13:2063. doi: 10.3390/v13102063, 34696495 PMC8540770

[43] Stoddard RA Gee JE Wilkins PP McCaustland K Hoffmaster AR. Detection of pathogenic Leptospira spp. through TaqMan polymerase chain reaction targeting the LipL32 gene. Diagn Microbiol Infect Dis. (2009) 64:247–55. doi: 10.1016/j.diagmicrobio.2009.03.014, 19395218

[44] Dubey JP Frenkel JK. Toxoplasmosis of rats: a review, with considerations of their value as an animal model and their possible role in epidemiology. Vet Parasitol. (1998) 77:1–32. doi: 10.1016/s0304-4017(97)00227-6 9652380

[45] Hosseini SA Abediankenari S Amouei A Sarvi S Sharif M Rezaei F . Seroprevalence of *Toxoplasma gondii* in wild rats (*Rattus rattus*) in northern Iran. Vet Med Int. (2021) 2021:6655696. doi: 10.1155/2021/6655696, 34136114 PMC8175164

[46] R Core Team. *R: A Language and Environment for Statistical Computing R Foundation for Statistical Computing*. Vienna: R Core Team (2021). Available online at: https://www.R-project.org/.

[47] Stevenson M Sergeant E Firestone S. *EpiR: Tools for the Analysis of Epidemiological Data (Version 2.0.65) [R Package]*. (2023). Available online at: https://CRAN.R-project.org/package=epiR.

[48] Lê S Josse J Husson F. FactoMineR: an R package for multivariate analysis. J Stat Softw. (2008) 25:1–18. doi: 10.18637/jss.v025.i01

[49] Jankowski G Adkesson MJ Saliki JT Cárdenas-Alayza S Majluf P. Survey for infectious disease in the south American fur seal (*Arctocephalus australis*) population at Punta San Juan, Peru. J Zoo Wildl Med. (2015) 46:246–54. doi: 10.1638/2014-0120.1 26056875

[50] Balcázar L Azócar-Aedo L Barrera V Meniconi G Muñoz V Valencia-Soto C. Detection of antibodies for pathogenic *Leptospira* in wild mammals and birds from southern Chile—first record of seropositivity in a guiña (*Leopardus guigna*). Animals. (2024) 14:601. doi: 10.3390/ani14040601, 38396569 PMC10886123

[51] Sepúlveda MA Seguel M Alvarado-Rybak M Verdugo C Muñoz-Zanzi C Tamayo R. Postmortem findings in four south American sea lions (*Otaria byronia*) from an urban colony in Valdivia, Chile. J Wildl Dis. (2015) 51:279–82. doi: 10.7589/2013-07-161, 25380367

[52] Greig DJ Gulland FM Kreuder C. A decade of live California Sea lion (*Zalophus californianus*) strandings along the Central California coast: causes and trends, 1991-2000. Aquat Mamm. (2005) 31:11–22. doi: 10.1578/AM.31.1.2005.11

[53] Reisfeld L Sacristán C Machado EF Sánchez-Sarmiento AM Costa-Silva S Ewbank AC . Toxoplasmosis and *Sarcocystis* spp. infection in wild pinnipeds of the Brazilian coast. Dis Aquat Org. (2019) 136:235–41. doi: 10.3354/dao03410 31724556

[54] Seguel M Muñoz F Paredes E Navarrete MJ Gottdenker NL. Pathological findings in wild rats (*Rattus rattus*) captured at Guafo Island, northern Chilean Patagonia. J Comp Pathol. (2017) 157:163–73. doi: 10.1016/j.jcpa.2017.07.006, 28942299

[55] Vieira AS Pinto PS Lilenbaum W. A systematic review of leptospirosis on wild animals in Latin America. Trop Anim Health Prod. (2018) 50:229–38. doi: 10.1007/s11250-017-1429-y, 28967042

[56] Torres FDA Borges ALDSB Kolesnikovas C Domit C Barbosa CB Carvalho-Costa FA . Pinnipeds carriers of pathogenic *Leptospira*: new data based on molecular characterization. Res Vet Sci. (2023) 155:62–8. doi: 10.1016/j.rvsc.2022.12.012, 36634544

[57] Buhnerkempe MG Prager KC Strelioff CC Greig DJ Laake JL Melin SR . Detecting signals of chronic shedding to explain pathogen persistence: *Leptospira interrogans* in California Sea lions. J Anim Ecol. (2017) 86:460–72. doi: 10.1111/1365-2656.12656, 28207932 PMC7166352

[58] Smith KM Karesh WB Majluf P Paredes R Zavalaga C Reul AH . Health evaluation of free-ranging Humboldt penguins (*Spheniscus humboldti*) in Peru. Avian Dis. (2008) 52:130–5. doi: 10.1637/8265-071007-Reg, 18459309

[59] Watson MK Langan JN Allender MC Cardeña M Cárdenas-Alayza S Adkesson MJ. Health assessment of guanay cormorant (*Phalacrocorax bougainvillii*) and Peruvian pelican (*Pelecanus thagus*) populations at Punta San Juan, Peru. J Zoo Wildl Med. (2021) 52:975–85. doi: 10.1638/2019-0119.1

[60] Acosta ICL Souza-Filho AF Muñoz-Leal S Soares HS Heinemann MB Moreno L . Evaluation of antibodies against *Toxoplasma gondii* and *Leptospira* spp. in Magellanic penguins (*Spheniscus magellanicus*) on Magdalena Island, Chile. Vet Parasitol Reg Stud Reports. (2019) 16:100282. doi: 10.1016/j.vprsr.2019.100282, 31027597

[61] Acosta ICL Chiebao DP Serafini PP Canani G Pena HFJ Heinemann MB . Analysis of free-living seabirds from Brazil as potential hosts of *Toxoplasma gondii* and serological investigation for antibodies against *Leptospira* spp. Vet Res Commun. (2025) 49:14. doi: 10.1007/s11259-024-10575-x 39560806

[62] Deem SL Merkel J Ballweber L Vargas FH Cruz MB Parker PG. Exposure to *Toxoplasma gondii* in Galapagos penguins (*Spheniscus mendiculus*) and flightless cormorants (*Phalacrocorax harrisi*) in the Galapagos Islands, Ecuador. J Wildl Dis. (2010) 46:1005–11. doi: 10.7589/0090-3558-46.3.1005, 20688714

[63] Campbell K Paparini A Gomez AB Cannell B Stephens N. Fatal toxoplasmosis in little penguins (*Eudyptula minor*) from Penguin Island, Western Australia. Int J Parasitol. (2022) 17:211–7. doi: 10.1016/j.ijppaw.2022.02.006, 35198375 PMC8850582

[64] Ploeg M Ultee T Kik M. Disseminated toxoplasmosis in black-footed penguins (*Spheniscus demersus*). Avian Dis. (2011) 55:701–3. doi: 10.1637/9700-030411-Case.1, 22312996

[65] Plowright RK Reaser JK Locke H Woodley SJ Patz JA Becker DJ . Land use-induced spillover: a call to action to safeguard environmental, animal, and human health. Lancet Planet Heath. (2021) 5:e237–45. doi: 10.1016/S2542-5196(21)00031-0, 33684341 PMC7935684

[66] Fan Y Hou Y Li Q Dian Z Wang B Xia X. RNA virus diversity in rodents. Arch Microbiol. (2024) 206:9. doi: 10.1007/s00203-023-03732-4 38038743

[67] Shehata AA Parvin R Tasnim S Duarte PM Rodriguez-Morales AJ Basiouni S. The hidden threat: rodent-borne viruses and their impact on public health. Viruses. (2025) 17:809. doi: 10.3390/v17060809, 40573400 PMC12197361

[68] Cortez V Canal E Dupont-Turkowsky JC Quevedo T Albujar C Chang TC . Identification of *Leptospira* and *Bartonella* among rodents collected across a habitat disturbance gradient along the inter-oceanic highway in the southern Amazon Basin of Peru. PLoS One. (2018) 13:e0205068. doi: 10.1371/journal.pone.0205068, 30300359 PMC6177132

[69] Darlan DM Mayasari E Hutagalung SV Kurniawan A Sinaga LA Pinem A . Molecular and serological detection of *Leptospira interrogans* among wild rats in flood-prone residential areas of Indonesia. Open Vet J. (2025) 15:437. doi: 10.5455/OVJ.2025.v15.i1.39, 40092209 PMC11910279

[70] Byers KA Lee MJ Patrick DM Himsworth CG. Rats about town: a systematic review of rat movement in urban ecosystems. Front Ecol Evol. (2019) 7:13. doi: 10.3389/fevo.2019.00013

